# Filling the gap in functional trait databases: use of ecological hypotheses to replace missing data

**DOI:** 10.1002/ece3.989

**Published:** 2014-02-25

**Authors:** Simon Taugourdeau, Jean Villerd, Sylvain Plantureux, Olivier Huguenin-Elie, Bernard Amiaud

**Affiliations:** 1 Agronomie et Environnement, UMR 1121, Université de LorraineVandoeuvre-lès-Nancy, F-54500, France; 2 Agronomie et Environnement, UMR 1121, INRAColmar, F-6800, France; 3 Agroscope Reckenholz-Tänikon Research Station ARTZurich, Switzerland; 4 Ecologie et Ecophysiologie Forestières, UMR 1137, Université de LorraineVandoeuvre-lès-Nancy, F-54500, France; 5 Ecologie et Ecophysiologie Forestières, UMR 1137, INRAVandoeuvre-lès-Nancy, F-54500, France

**Keywords:** Functional diversity, imputation methods, LEDA database, missing data, plant functional trait

## Abstract

Functional trait databases are powerful tools in ecology, though most of them contain large amounts of missing values. The goal of this study was to test the effect of imputation methods on the evaluation of trait values at species level and on the subsequent calculation of functional diversity indices at community level using functional trait databases. Two simple imputation methods (average and median), two methods based on ecological hypotheses, and one multiple imputation method were tested using a large plant trait database, together with the influence of the percentage of missing data and differences between functional traits. At community level, the complete-case approach and three functional diversity indices calculated from grassland plant communities were included. At the species level, one of the methods based on ecological hypothesis was for all traits more accurate than imputation with average or median values, but the multiple imputation method was superior for most of the traits. The method based on functional proximity between species was the best method for traits with an unbalanced distribution, while the method based on the existence of relationships between traits was the best for traits with a balanced distribution. The ranking of the grassland communities for their functional diversity indices was not robust with the complete-case approach, even for low percentages of missing data. With the imputation methods based on ecological hypotheses, functional diversity indices could be computed with a maximum of 30% of missing data, without affecting the ranking between grassland communities. The multiple imputation method performed well, but not better than single imputation based on ecological hypothesis and adapted to the distribution of the trait values for the functional identity and range of the communities. Ecological studies using functional trait databases have to deal with missing data using imputation methods corresponding to their specific needs and making the most out of the information available in the databases. Within this framework, this study indicates the possibilities and limits of single imputation methods based on ecological hypothesis and concludes that they could be useful when studying the ranking of communities for their functional diversity indices.

## Introduction

Advances in ecological research, combined with the increasing power of statistical analyses and computers, allow researchers to study more and more species under an increasingly wide range of environmental conditions (Spiegelberger et al. 2012). Ecological studies on plant community assemblages usually rely on large amounts of data compiled in databases, linking community assemblages, and environmental conditions data with data about the functional traits of the species. Such databases are crucial for improving our understanding of the effects of global changes, like the loss of biodiversity or climate change, on the biosphere (Kattge et al. 2011). This because on one hand, important plant functional traits are driven by environmental conditions (de Bello et al. 2005; Louault et al. 2005; Ackerly and Cornwell 2007; Ordoñez et al. 2009), and on the other hand, plant functional traits influence ecosystem functions, such as primary productivity and nutrient cycling (Mokany et al. 2008; Klumpp and Soussana 2009; de Bello et al. 2010).

Standardized protocols are available for the measurements of plant traits in the field (Cornelissen et al. 2003; Pérez-Harguindeguy et al. 2013), and these measurements are now collected in large, well-structured databases (Kleyer et al. 2008; Kattge et al. 2011) accessible to the scientific community. However, plant trait databases contain a lot of missing data and probably will continue to for a long time because of the labor-intensive nature of collecting well-informed, standardized data, and because studies with different aims are usually interested in different traits. It is therefore unrealistic to expect complete knowledge of a large number of species from various ecosystems. For instance, in the large database of the TRY initiative (Kattge et al. 2011), 39.1% of trait values concerned only four traits (specific leaf area, vegetative height, leaf dry matter content, and seed mass as 13.2%, 10.0%, 8.7%, and 7.2%, respectively). These four traits are frequently the best documented, and even for them, the percentage of missing data is high. For instance, in the LEDA database (Kleyer et al. 2008); status in 2011) among the 8195 registered species, only 2019 species have information on specific leaf area (75% missing), 1730 on leaf dry matter content (78% missing), 2492 on seed mass (69% missing), and 2893 for vegetative height (64% missing). Species with missing data are not generally the most dominant species observed in floristic relevés. Nevertheless, these missing data limit the optimal use of plant trait databases, as functional diversity indices, for instance, need to be calculated without missing values (Mason et al. 2005; Villeger et al. 2008).

An option still used to deal with these missing data is to delete species with missing data for the calculation of diversity indices (Lin et al. 2011). The obvious drawback is that it may introduce bias in the range of species retained for calculation and considerably reduce the dataset, consequently limiting the statistical power of any forthcoming analysis. Garnier et al. (2004) suggested that this deletion is acceptable for estimation of the community-weighted mean trait value (CWM) as long as it only concerns the minor species. They indicated that the deletion of minor species should not exceed 20% of the total biomass of the community. Indeed, if the value of a plant trait does not vary widely between species of a community, the weighted mean trait value of the community can be calculated with species that make up 80% of the total biomass of the communities. The additional effort required to sample species traits would not be worthwhile in terms of exactness (Pakeman and Quested 2007). However, exploring the effects of environmental constraints on plant community structure or the role of functional diversity in ecosystem processes without taking minor species into consideration could be misleading (Walker et al. 1999), as minor species can have a significant effect on ecosystem function (Boeken and Moshe 2006).

Another option used in some studies is to replace the missing data using an imputation method. In statistics, imputation is the process of replacing missing data with substituted values (Nakagawa and Freckleton 2008). Imputation can be simple: Missing data can be replaced by the mean or the median of the available trait values, as implemented in the studies of Gunton et al. (2011) and Fried et al. (2012). However, such simple imputation methods do not take the functional differences between species into account.

A third option, that is only relevant for functional diversity indices calculated from several traits, is to use the Gower distance and project the distance with a Principal Coordinate Analysis (Villeger et al. 2008; Mouillot et al. 2011). The Gower distance can be computed with some missing data (Gower 1971), and the PCoA allows projection of a distance matrix on several axes, the axes being then used as functional traits. This method assesses the functional spaces, but the trait information gets lost and only multivariate approaches can be used.

The problem of missing values in large matrices exists in a wide range of fields, and advanced mathematical methods of imputation to deal with it have been developed, like multiple imputation (Schafer and Graham 2002; Van Buuren et al. 2006; Van Buuren 2007; Azur et al. 2011). Multiple imputation is a Monte Carlo technique in which the missing values are replaced by *m *>* *1 imputed values. Each of the imputed complete datasets is analyzed by standard methods, and the results are combined to produce estimates and confidence intervals that incorporate missing data uncertainty (Nakagawa and Freckleton 2008). We did not find any utilization of multiple imputation on functional trait databases. For the utilization of more advanced missing data imputation on functional trait databases, we only found the study of Shan et al. (2012) that recently tested another type of method: The hierarchical probabilistic matrix factorization coupled with phylogenetic information to replace missing values in plant trait databases. Functional proximity between species (Westoby et al. 2002; Diaz et al. 2004) and relationships between traits (Wright et al. 2004, 2006) could also be used for imputation, making a comprehensive use of the information available in the trait database. An alternative method to deal with missing functional trait data without deleting species and taking functional relationships between species and/or traits into account would therefore improve the use of functional trait databases.

The aim of this study was to test imputation methods that integrate knowledge of relationships between species, but uses simple mathematics to impute missing data to calculate functional diversity indices based on functional trait databases. First, we tested the effects of several imputation methods on the evaluation of the trait values at the species level, using different levels of missing data and a range of functional traits with varying distribution. In a second step, the effects of these methods on the calculation of functional diversity indices at the community level were assessed for grassland communities.

## Materials and Methodology of Imputation of Missing Data

### Selection of two subdatabases without missing data and insertion of missing data

**Figure 1:**
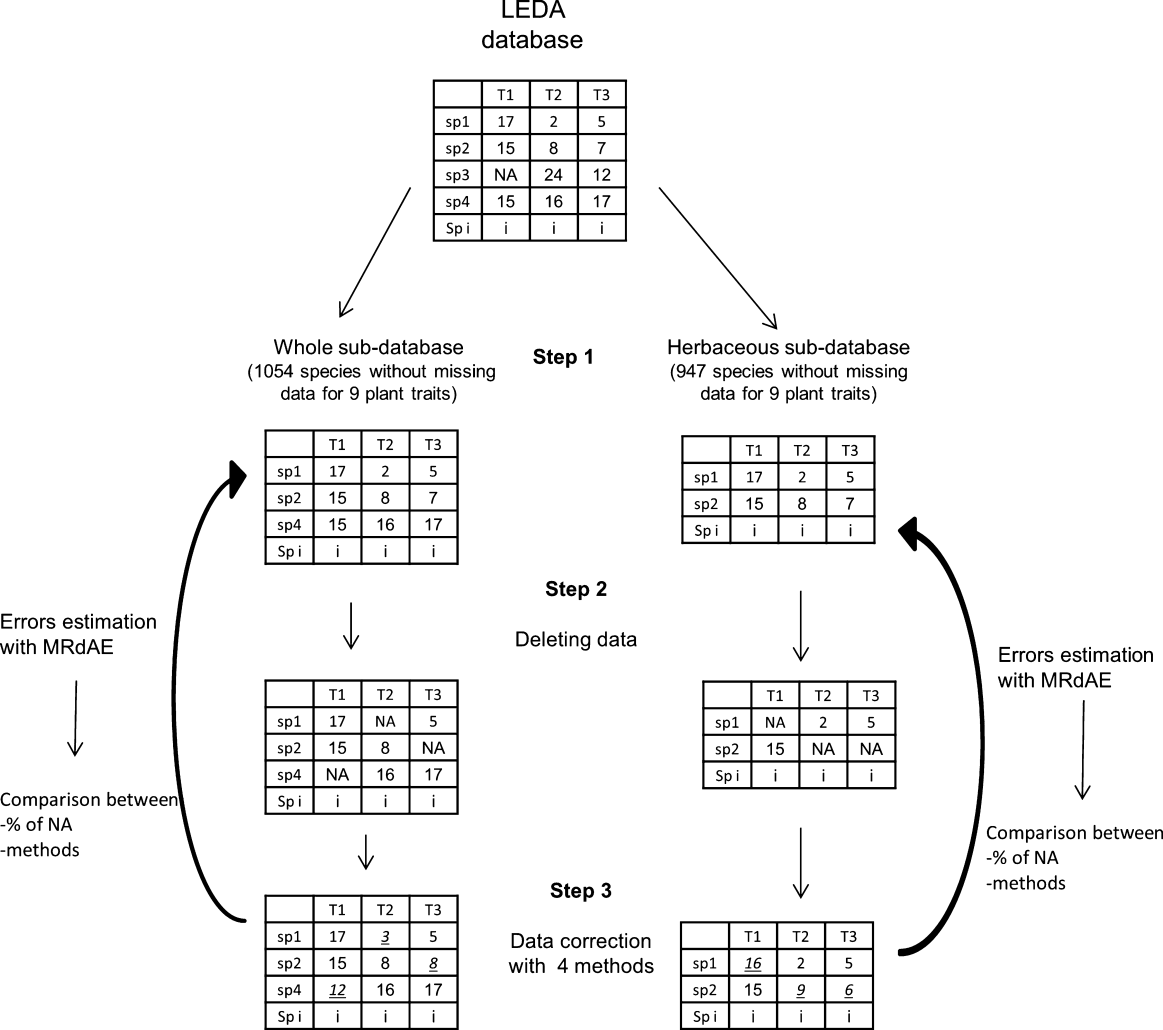
General procedure of estimation of errors for the imputation of missing data Step 1) creation of two trait subdatabases, one with no species filters and one only with herbaceous species; Step 2) missing data were inserted with 10 different percentages from 1% to 46%; the insertion was made 100 times per percentage of missing data (2000 different subdatabases were created this way); Step 3) these missing data were replaced using five different methods (10,000 corrected datasets were created this way); Step 4) errors induced by the imputation were estimated by comparison between the original database and the corrected one; Step 5) the error estimations were then compared between the different percentages of missing data for each method and between methods.

Initially, only the average trait values of the species in the LEDA database (Kleyer et al. 2008) were used. A total of 1054 herbaceous and ligneous plant species with no missing data for nine continuous traits were found in the database and retained to establish the “whole subdatabase” (Fig. 1 – step 1). These traits were vegetative height (H), reproductive height (RH), seed mass (SM), seed shape (SS), seed number per plant (SNP), specific leaf area (SLA), leaf dry matter content (LDMC), leaf mass (LM) and leaf surface (LS).

Within this subdatabase, the distribution of the trait values was similar for the vegetative height (H), the RH, LM, LS, SS, SM, and SNP. For these seven traits, most values were low with few extreme high values. The presence of a few tree species in the database is one reason for the unbalanced distribution of some traits. The distribution of the LDMC and the SLA values was close to a Gaussian distribution.

We also used a subdatabase with only herbaceous species to assess how strongly the error induced by the imputation methods depended on the distribution of the trait values in the database (Fig. 1 – step 1). This second subdatabase was set by eliminating the species with a vegetative height greater than 2 m and of the Raunkiaer types “phanerophyte” or “chamaephyte” to eliminate trees and shrubs from the whole subdatabase. In this second subdatabase, called “herbaceous subdatabase,” 947 species were documented with the same nine plant traits as for the whole subdatabase. The vegetative height (H) and the reproductive height (RH) had a normal distribution in the herbaceous subdatabase.

In these two subdatabases, missing data were deliberately inserted by randomly deleting existing values (Fig. 1 – step 2). Each existing value in the subdatabase had a given probability of being deleted. Ten different probabilities of deletion were applied (from 0.01 to 0.46 with an interval of 0.05; same probability for all values of the subdatabase at each step), yielding large differences in the level of missing data insertion. For each level of missing data, the random deletion was made 100 times. The deleted values could be different for each simulation. One thousand different versions of the two subdatabases were created (10 levels of deletion probability × 100 random deletions).

### Imputation methods

Five imputation methods were tested (Fig. 1 – step 3). These included two simple mathematical methods (“average” and “median”), as well as three methods that to our knowledge have not yet been implemented for imputation in functional trait databases: two methods based on ecological hypotheses and thereafter called the “dissimilarity” and the “relationships” methods, as well the multivariate imputation by chained equations (a multiple imputation method; Azur et al. 2011; Van Buuren and Groothuis-Oudshoorn 2011).

The two single mathematical imputation methods consisted of either replacing the missing data with the average trait value (average method) or by the median value of all species with documented values (median method). These methods have already been used in literature (Gunton et al. 2011; Fried et al. 2012). In these two methods, the missing values of trait T_*i*_ for the species S_*i*_ to S_*j*_ are all replaced with the same value, without using the information that could be available from other traits.

The dissimilarity imputation method is based on the functional proximity between species. This method relies on the hypothesis that species with the same functional strategy have a similar set of functional traits (Westoby et al. 2002; Diaz et al. 2004). To replace the missing data of the trait T_*j*_ of the species S_*i*_, the Gower dissimilarity (Gower 1971) between S_*i*_ and the other species is calculated based on the other traits. The species showing high similarity with S_*i*_ are then selected, and the median of their trait values for T_*j*_ is computed and used to evaluate the missing value T_*ji*_. We chose a Gower dissimilarity coefficient of 0.05 as threshold for species with high similarity. The Gower dissimilarity can be computed with missing data, so the presence of other missing data would not disrupt replacement of the missing data.

The relationship imputation method depends on the existence of relationships between plant traits (Wright et al. 2004, 2006). For each trait (T_*j*_), the dataset is split in two matrices, according to the presence or absence of missing data for T_*j*_: the first matrix containing all species with no missing data for T_*j*_ and the second matrix with all species with missing data for T_*j*_. On the first matrix, a statistical model explaining T_*j*_ using the other traits is created by a stepwise regression. Then, this model is used to estimate the missing data in the second matrix. When, in a few cases, the value of another trait T_2_ entering in the model for the estimation of the missing value T_1*i*_ was also missing for S_*i*_, we replaced the missing value of T_2*i*_ with the median trait value of T_2_. The occurrence of such a replacement of the missing value of another trait increased with increasing percentage of missing data.

R scripts (R Development Core Team 2013) used to implement the two methods based on ecological hypothesis are available by request to the authors.

The four methods presented above perform simple imputation (each missing entry is replaced by a single imputed value). The resulting imputed dataset therefore contains genuine as well as simulated data. With such methods, the uncertainty associated with imputed data is lost and cannot be propagated to the analyses to be applied on the imputed dataset. In contrast, the objective of the multiple imputation approach is to handle missing data in a way resulting in valid statistical inference, rather than to predict missing values as close as possible to the true ones (Rubin 1996). Concretely, m different imputed values are generated for each missing entry, leading to m different imputed datasets. Analyses (here functional diversity indices computation) are then carried out on each imputed dataset and pooled to produce estimates and confidence intervals that incorporate missing data uncertainty. We here also used a method of multiple imputation: the Multivariate Imputation by Chained Equations (MICE, Azur et al. 2011). The MICE method was computed using the “mice” package of R (Van Buuren and Groothuis-Oudshoorn 2011). This method of imputation uses predictive mean matching with five imputations. For the species level, the average of the 5 values imputed was used to replace the missing value.

### Comparison of the errors between methods and percentage of missing data

For each simulation, we compared the original plant trait value (To)_*ij*_ and the value after replacement (Tr)_*ij*_ (Fig. 1 – step 4).

The quality of the replacement was evaluated by an indicator independent of the number of missing data: a modified median relative absolute error (MRdAE) of the imputed values (MRdAE = median(abs [(To−Tr)_*ij*_]/median[To]_*ij*_).The modification as compared to the MRdAE used in Hyndman and Koehler (2006) is that the denominator is equal to the median of the original value instead of abs (To[*ij*]−median [To(*j*)]) Indeed, we wanted to assess the deviation from the original value of the functional trait rather than to compare two variables.

In our study, this indicator is more suitable than other common error measures such as the root-mean-square error for two major reasons. First, the MRdAE does not depend on the number of estimated values (i.e., the rate of missing values). Secondly, it is less sensitive to outliers (Hyndman and Koehler 2006).

A Kruskal–Wallis nonparametric analysis of variance (ANOVA) test was realized on the MRdAE between the 10 different probabilities of missing data for each trait, for each method, and on the two subdatabases. This analysis was made 90 times (nine traits × five methods × two subdatabases). When the Kruskal–Wallis *P* value is not significant, it means that for a given subdatabases, the replacement method creates the same error during the replacement irrespective of the percentage of data that were missing and replaced. On the contrary, when the Kruskal–Wallis *P* value is significant, the accuracy of the method depends on the percentage of missing data (Fig. 1 – step 5).

We also compared the MRdAE between the four different methods using a Kruskal–Wallis nonparametric ANOVA. The comparison was made for each trait on each dataset but without separating the levels of missing data (analysis run 18 times: nine traits × two datasets; Fig. 1 – step 5). A multiple comparison test after Kruskal–Wallis (ad hoc test) was conducted (Siegel and Castellan 1988).

## Results of Imputation Methods at the Species Level

### Differences between the imputation methods on the whole subdatabase

The average method was the least accurate (higher MRdAE) for all the traits studied. The MRdAE of the average method was highly variable between traits, from 0.25 for the SLA to 44.62 for the SNP (Table 1). The median method was less accurate than the dissimilarity method for all traits except for the SNP, but it was more accurate than the relationships method except for the SLA and the LDMC. The relationships method was therefore in most case less accurate than the dissimilarity method. For the SLA and LDMC, the MRdAE of the five methods was low with similar values (around 0.24; Table 1). For the other traits (H, RH, LM, LS, SS and SNP), the MRdAE of the single imputation methods was higher than for the SLA and the LDMC and ranged from 0.49 (RH with dissimilarity method) to 45.00 (SNP with average method). The differences between the methods were also more distinct with these traits than with the SLA and the LDMC (Table 1). The MICE method was more accurate than all other methods for all traits except for the specific leaf area.

**Table 1:** Median relative absolute error (MRdAE) for each imputation method in the two subdatabases, averaged over all percentages of missing data (1–46%). A Kruskal–Wallis ANOVA and its ad hoc test were conducted to test the effect of the different methods on the MRdAE. A lower MRdAE means less error due to the imputation of missing values. The letters in a column correspond to the results of the ad hoc test

	H		LDMC		LM		LS		RH		SM		SNP		SS		SLA	
Methods	MRdAE	Adhoc	MRdAE	Adhoc	MRdAE	Adhoc	MRdAE	Adhoc	MRdAE	Adhoc	MRdAE	Adhoc	MRdAE	Adhoc	MRdAE	Adhoc	MRdAE	Adhoc
Whole database																		
Average	1.158	a	0.276	a	4.570	a	4.203	a	1.061	a	7.561	a	44.623	a	0.791	a	0.253	a
Median	0.531	d	0.262	d	0.918	d	0.941	d	0.516	d	0.904	d	1.168	d	0.515	c	0.239	d
MICE	0.253	b	0.235	b	0.164	b	0.192	b	0.259	b	0.242	b	0.283	b	0.426	b	0.241	b
Dissimilarity	0.495	c	0.252	c	0.779	c	0.834	c	0.477	c	0.798	c	1.356	c	0.480	b	0.231	c
Relationships	0.735	e	0.242	e	1.685	e	1.210	d	0.770	e	4.123	e	44.263	a	0.647	d	0.232	e
Herbaceous database																		
Average	0.590	a	0.281	a	4.241	a	3.774	a	0.528	a	5.342	a	38.180	a	0.776	a	0.250	a
Median	0.512	d	0.258	d	0.926	d	0.932	d	0.483	d	0.893	d	1.158	d	0.516	d	0.233	cd
MICE	0.209	b	0.239	b	0.159	b	0.194	b	0.201	b	0.303	b	0.257	b	0.421	b	0.238	b
Dissimilarity	0.306	c	0.242	c	0.625	c	0.641	c	0.295	c	0.763	c	1.504	c	0.460	c	0.230	c
Relationships	0.226	e	0.249	e	1.597	e	1.185	e	0.223	e	3.354	e	34.725	e	0.629	e	0.233	d

H, vegetative height; LDMC, leaf dry matter content; LM, leaf mass; LS, leaf surface; MICE, Multivariate Imputation by Chained Equations; RH, reproductive height; SM, seed mass; SNP, seeds number per plant; SS, seed shape; SLA, specific leaf area.

### Differences between methods on the herbaceous subdatabase: effect of the trait distribution

The use of the herbaceous subdatabase affected the results only for the vegetative height (H) and the reproductive height (RH; Table 1). The distribution of these two traits was unbalanced for the whole subdatabase and balanced for the herbaceous subdatabase (results not shown). The MRdAE of the five imputation methods was lower for the herbaceous subdatabase in comparison with the whole subdatabase for these two traits. The minimal MRdAE of the single imputation methods was less when working with herbaceous plants only (and therefore with a balanced distribution of the traits) rather than with the whole subdatabase (0.22 and 0.48, respectively; Table 1). Moreover, the relationships method was more accurate than the dissimilarity method for H and RH when using the herbaceous subdatabase rather than the whole subdatabase. No difference in accuracy ranking of the relationships and the dissimilarity methods was found between the whole subdatabase and the herbaceous subdatabase for the other traits because their distributions remain unchanged. In comparison with the whole subdatabase, the accuracy of the MICE methods for the H and the RH was higher with the herbaceous subdatabase (MRdAE 0.21 for H and MRdAE of 0.20 for RH).

### Effect of the level of missing data

The average method was not affected by the percentage of missing data on the two subdatabases except for the SNP with the herbaceous subdatabase (Table 2). The median method was only affected by the percentage of missing data for the SNP on the two subdatabases and the SM in the herbaceous subdatabase. The dissimilarity method was affected for seven traits in the herbaceous subdatabase and only for four traits in the whole subdatabase. The relationships method was the most sensitive to the level of missing data. This method was affected by the percentage of missing data for five traits for the herbaceous subdatabases and eight traits for the whole subdatabase (Table 2). The MICE method was affected by the percentage of missing data for six traits on the whole subdatabase and seven for the herbaceous subdatabase.

**Table 2:** Effect of percentage of missing data on the MRdAE (median relative absolute error) for the four methods applied to the two subdatabases. For each method, a one-way Kruskal–Wallis test was conducted to test the effect of the percentage of missing data on the MRdAE. The *P* values are presented in the table for each method and each trait

	Traits								
Methods	H	LDMC	LM	LS	RH	SM	SNP	SS	SLA
Whole subdatabase									
Average	0.55	0.44	0.24	0.11	0.34	0.38	0.06	0.48	0.38
Median	0.72	0.22	0.42	0.97	0.33	0.55	**0.01**	0.66	0.37
MICE	0.22	**0.00**	**0.00**	**0.00**	0.11	**0.00**	0.53	**0.01**	**0.00**
Dissimilarity	0.46	0.87	**0.00**	0.11	0.25	**0.00**	**0.01**	**0.01**	**0.00**
Relationships	**0.00**	**0.00**	**0.00**	**0.00**	**0.02**	**0.00**	0.40	**0.00**	**0.00**
Herbaceous subdatabase									
Average	0.69	0.55	0.07	0.07	0.47	0.32	**0.01**	0.80	0.95
Median	0.89	0.03	0.00	0.19	0.20	**0.01**	**0.00**	0.46	0.89
MICE	**0.00**	**0.00**	**0.00**	**0.00**	**0.00**	0.36	1.00	**0.01**	**0.00**
Dissimilarity	**0.00**	**0.00**	**0.00**	**0.00**	**0.00**	0.52	**0.00**	**0.00**	0.07
Relationships	**0.00**	0.31	**0.00**	**0.00**	**0.00**	**0.00**	0.36	0.39	0.16

H, vegetative height; LDMC, leaf dry matter content; LM, leaf mass; LS, leaf surface; MICE, Multivariate Imputation by Chained Equations; RH, reproductive height; SM, seed mass; SNP, seeds number per plant; SS, seed shape; SLA, specific leaf area.

Significant *P* values (*P* < 0.05) are in bold.

## Discussion of the Accuracy of the Imputation Methods

The results show that at the species level, the most accurate imputation method is not the same for all traits and in all cases, but one of the methods based on ecological hypothesis (dissimilarity and relationships methods) was always the most accurate among the single imputation methods. The relatively low MRdAE values found with at least one of the ecological methods for all the traits included in this study, particularly with the herbaceous subdatabase, indicate the potential of these methods for the replacement of missing values prior to the calculation of functional diversity indices.

Among the single imputation methods, the dissimilarity method is the most accurate when the trait distribution is unbalanced, as in leaf mass or leaf surface (Table 1). In this situation, the median method is almost as accurate as the dissimilarity method, whereas the relationships method does not perform well on very unbalanced traits (like SNP) because the multilinear model is strongly governed by extreme values. However, when the trait distribution is more balanced, the accuracy of the relationships method is similar (LDMC and SLA for the two subdatabases) or slightly better than that of the dissimilarity method (H and RH for the herbaceous subdatabase).

The multivariate imputation in chained equations was the most accurate method for the unbalanced trait (H, RH, SM, SNP, SS, LM, and LS). For the SLA, the MICE method induces slightly more error that the ecological based methods. For the other balanced traits (LDMC and H or RH for herbaceous subdatabase), the difference between MICE and the relationships method was low. In the MICE method, the correction model can be adapted to the distribution of the variable (Azur et al. 2011; Van Buuren and Groothuis-Oudshoorn 2011), so that the traits with an exponential distribution are well corrected. This explains the higher accuracy of the MICE method on the unbalanced functional traits.

Comparing the results obtained with the two subdatabases, the error was lower when the traits had a balanced distribution (with the relationships method) than when the traits had an unbalanced distribution. It seems better to choose a subdatabase with balanced traits distribution by, for example, only using herbaceous species for grassland studies rather than all type of plants species. Traits' distributions explain the differences in accuracy observed between the single imputation methods, the traits, and the subdatabases. The key parameter to choose the adequate imputation method is thus the distribution of the value of the trait in the dataset. This also indicates that applying a transformation method to improve the distribution of the trait values prior to using a imputation method could be useful in improving the quality of the replacement.

The objectives and methods of the study should also be considered when choosing the imputation method. For instance, replacing the missing data using distances between species (dissimilarity method) would not be an appropriate choice for a study on functional distance between species, as functional distance would then be underestimated. Functional distance between species is often used to classify species into groups or to calculate some functional diversity indices (Rao 1982; Mouchet et al. 2008).

**Figure 2:**
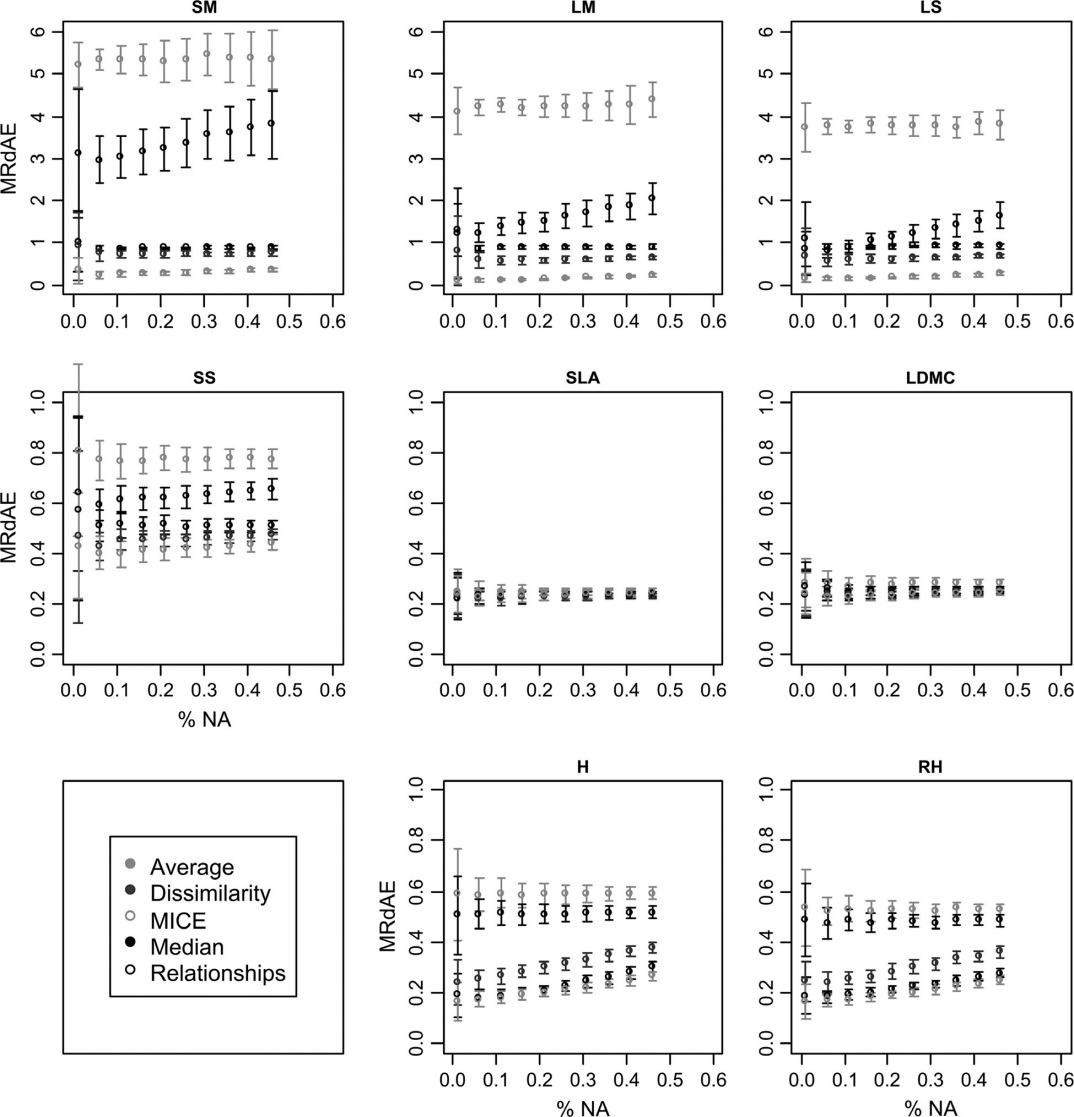
Evolution of the MRdAE of five imputation methods in the herbaceous subdatabase with different percentages of missing data for eight traits (SM, seed mass; LM, leaf mass; LS, leaf surface; SS, seed shape; SLA, specific leaf area; LDMC, leaf dry matter content; H, vegetative height; RH, reproductive height). The mean of 100 simulations ± the standard deviation is shown for each percentage of missing data.

The relationships method is very sensitive to the percentage of missing data (Table 2). This could be due to the replacement of missing values of other traits by the median value of these traits that was needed for the creation and the utilization of the multilinear models. The negative effect of these replacements on the accuracy of the estimated values increased with an increasing percentage of deleted data (Fig. 2). The dissimilarity method is less affected by the percentage of deleted data. Indeed the metric use to calculate the dissimilarity, the Gower dissimilarity coefficient is able to deal with missing data up to a certain threshold. Nevertheless, the Gower dissimilarity cannot be calculated between two species if no trait is documented for both species, and so the correction would not be possible if missing data are too numerous. In the hierarchical probabilistic matrix factorization method tested by Shan et al. (2012), phylogenetic information from an independent source is used to create groups of plants with trait values of reduced variability and the mean of the existing trait values is used to predict missing values within such groups. Shan et al. (2012) showed that this method is satisfactory to predict trait values when information at the genus level is available. Instead of phylogenetic information from another database, the method considered here uses relationships between traits, and hence, all the information available within the trait database and the mathematics involved are simpler. It is thus comparatively straightforward to apply. On the other hand, while the method propose by Shan et al. (2012) needs only at least one trait value per plant, the method considered here requires several traits per plant/species to be documented.

In the different studies on missing data and imputation, the distribution of the missing data is a key parameter (Schafer and Graham 2002; Nakagawa and Freckleton 2008). Three different types of distribution of the missing data are described in the literature: missing completely at random (MCAR), missing at random (MAR), and missing not at random (MNAR). In functional trait databases, missing data will seldom be MCAR, because the missingness is related to the frequency of the species and their abundances. Indeed, the probability that a species was measured and implemented in the functional trait database is higher if this species is frequent and/or abundant than if it is seldom. Nevertheless, we found no relationships between the functional trait values of the nine traits and the frequency and average abundance of the species in our relevés dataset used for the calculation of functional diversity indices (below). Therefore, the missingness of the data in the original trait database was probably not related to the value of the traits. Regarding the trait values, the missingness produced by our random deletion was therefore similar to the missingness in the original database.

Our results present the error induced by different methods of imputation at the species level. Functional trait databases are often used to compute functional diversity indices of communities, and it is therefore necessary to evaluate the effects of imputation of missing data at community level.

## Effects of the Imputation Methods for the Calculation of Functional Diversity Indices

### Material and methods

We tested the effect of missing data and the difference between the methods of imputation on the computation of three functional diversity indices at the community level using grassland communities' data. These indices were the community-weighted mean value of the trait (functional identity), its functional range, as well as its functional divergence. The functional range of the traits (difference between the minimum and the maximum) is important to understand the rules of plant community assemblage (Petchey and Gaston 2002, 2006; Mouchet et al. 2010). The functional divergence corresponds to the repartition of the abundance regarding functional identity within a plant community (Mason et al. 2005; Mouchet et al. 2010). We chose the functional divergence index proposed by Schleuter et al. (2010) among the several indices available for the calculation of functional divergence.

The functional traits were extracted from the LEDA trait database (Kleyer et al. 2008), Fig. 3A). We limited the trait selection to four traits (SLA, SM, H, and LDMC) often used in grassland studies. The SLA, H, and SM are, for instance, the traits proposed on the leaf-height-seed (LHS) model of Westoby et al. (2002), which is useful to assess the live strategy of the species. Moreover, LDMC and SLA are important traits in the leaf economic spectrum and are often linked with ecosystem function.

**Figure 3:**
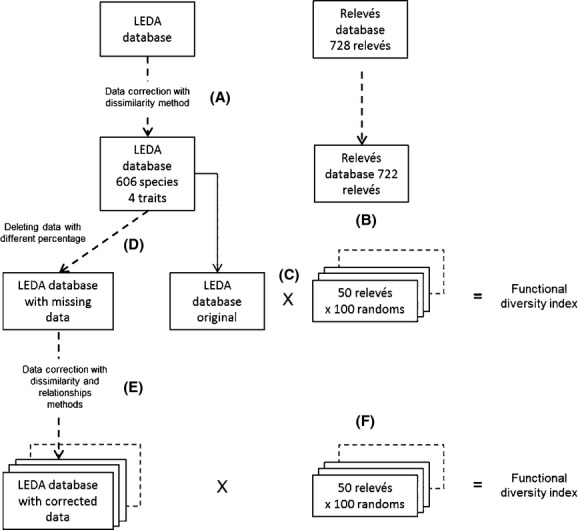
General procedure the assessment of the effects of the imputation methods for the calculation of functional diversity indices. (A) a database without missing data was created from the LEDA database (four traits for 526 species; some replacement of missing values by the dissimilarity methods where necessary); (B) 50 relevés were randomly selected from a large set of relevés (this process was repeated 100 times); (C) 50 relevés and the trait database were crossed and functional diversity indices were computed; (D) missing data were inserted in the trait database with several percentages; (E) missing data were replaced with the dissimilarity and the relationships methods; (E) these corrected databases were crossed with the 50 relevés, and functional diversity indices were computed; (F) the indices computed from database without missing values were compared to the indices computed from the databases with replaced missing values using a Pearson's correlation test.

The grassland botanical relevés originated from three datasets: one from the Swiss Alps (Peter et al. 2008a,b2008b), one from the Vosges mountains in northeastern France (Plantureux and Thorion 2005), and another from a broader range of regions in France from Atlantic to continental conditions (Michaud et al. 2012). The grassland relevés used to represent a large gradient of ecologic filters (climatic and agricultural management).

Our first attempt involved only relevés where all the species have a value for the four traits in the database. However, only four relevés fell within this constraint. Therefore, to start our test with enough data for the species present in the relevés, the missing trait values in the LEDA database had to be imputed. Imputation was used on 20 species for H (3% of the data), on 136 species for LDMC (22%), 69 species for SM (11%), and 96 species for SLA (15%). The dissimilarity method was used, as it proved satisfactory for the H, LDMC and the SLA in the first part of the study. SM, for which the dissimilarity method was less accurate, had only 11% of missing values. Species unidentified in the surveys and species with missing data for the four traits were omitted. Only the relevés where the abundance of these unidentified species was inferior to 5% of the total abundance were kept. After these modifications, 722 relevés were available with 606 species.

The use of the dissimilarity imputation before the insertion of missing data induced some circularity in the evaluation of the imputation method. However, we think that the circularity is low. This circularity would be very problematic if a trait value was imputed twice the same way. In our work, this probability of double imputation is very low. Indeed, the imputation of one value depends on all the different trait values of the other species and also the missing data on the entire functional trait database. Indeed, the calculation of the dissimilarity would differ between two calculations if the missing data are not exactly on the same trait values. The selection of the close species in the dissimilarity method is related to the calculation of the Gower dissimilarity and so to the distribution of missing data in the functional trait database. Secondly, the calculation of the median of the trait value of the close species depends also on the presence of missing data for the functional trait value of these species.

Different other option could have to use: only use the dominant species in the survey (80% of the abundance) or virtually assemble species. The use of only dominant species would leave out the minor species. If we only interest of the dominant species, the percentage of missing data would be quite low and so the necessity of imputation would be less important.

The creation of artificial species assemblages with only species having a value for the four traits in the database would have yield unrealistic differences in functional diversity indices of the communities, because the majority of these species would have been common and thus ubiquist species. Thus, we consider that replacing some missing trait values in true communities to create a complete database as comparison point for our study was the most appropriate option.

Among these 722 relevés, for each simulation, we randomly selected 50 different relevés. This random selection was made 100 times to have 100 sets of 50 plant communities (Fig. 3B). Each set of relevés was crossed with the functional trait database.

We deliberately inserted missing data in the trait database, by randomly deleting some trait values (Fig. 3D), and so created datasets with different percentages of missing data (1%, 5%, 10%, 20%, 30%, 40%, and 50%). For each percentage, the insertion of missing data was made 100 times (one insertion per set of 50 communities). These missing data were then replaced using the dissimilarity, the relationships, or the MICE method (Fig. 3E) to create functional trait databases with imputed data. We did not examine imputation by the median or the average on the calculation of functional diversity indices, because at the species level, one of the two ecological methods was always better or as good as the two mathematical methods (Table 2). The 50 communities were crossed with these trait databases with different percentages of replaced missing data, and functional diversity indices were computed (Fig. 3F). For the MICE method, the functional diversity indices were computed for each of the five imputations and the average value of these five estimations of the diversity indices was used for the comparison. The indices calculated from the values of the datasets with imputed values were compared to those calculated from the original database (without missing data) using a Pearson's correlation test. From this comparison, we assessed the effect of replacing missing data on the ranking between the functional diversity indices of 50 grasslands. The *P* value was calculated for each correlation between the two rankings for 100 sets of 50 grasslands. In most studies on functional diversity, the ranking between communities is more important than the absolute value of the functional diversity. We thus focused on the effect of replacing missing data on this ranking. For the discussion, we use the following threshold: If the correlation *P* value was not significant for five or more of the 100 sets of communities, the results obtained by the imputation methods were considered unsuitable (by similitude with significant threshold at 5%). The percentage of missing data for which this threshold was exceeded was estimated by linear estimation between the simulations with the different levels of missing data.

We also conducted the simulation on the ranking of the communities for their functional diversity indices after deleting the species with a missing value (deletion option, also known as “complete-case analysis”).

### Results on the effect of imputation methods on functional diversity indices

#### Community-weighted mean (functional identity)

**Figure 4:**
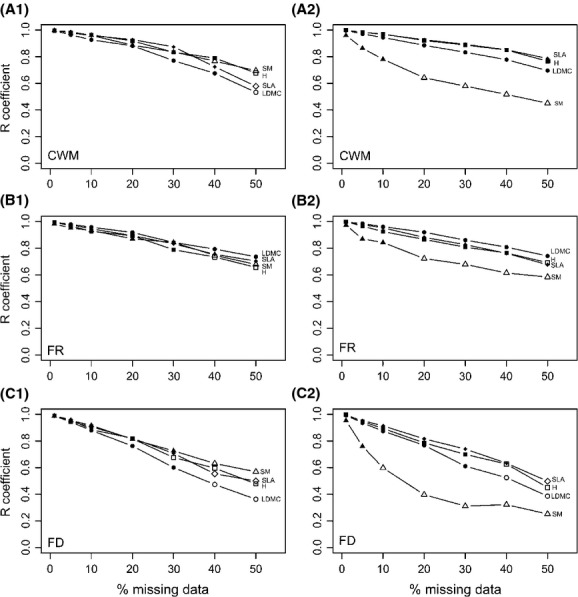
Effect of percentage of missing data on the R Pearson's coefficient between functional diversity indices calculated without missing data and with imputed data (A) on the community-weighted mean (A1 with the dissimilarity method, A2 with the relationships method); (B) on the functional range (B1 with the dissimilarity method, B2 with the relationships method); (C) on the functional divergence index (C1 with the dissimilarity method, C2 with the relationships method). The formats of the dots represent the functional trait used for the computation of the indices: Triangle for the seed mass (SM), diamond for the specific leaf area (SLA), circle for the leaf dry matter content (LDMC), and square for the vegetative height (H). Full dots represent levels of percentage of missing data where at least 95% of the correlations were significant (*P* value < 0.05). Empty dots represent the simulation where less than 95% of the correlations were significant (*P* value < 0.05).

When the missing data were replaced using the dissimilarity method, the ranking between grasslands based on the community-weighted mean (CWM) values was not affected by the percentage of missing data until more than 40% of the data were missing for SLA, LDMC, and H. For the CWM of SM, the ranking was impacted by the imputation from 31% of missing data upwards (Table 3). The R Pearson's coefficients were slightly higher for H and SM than for SLA and LDMC (Fig. 4A1). When the missing data were replaced using the relationships method, the ranking of grasslands based on the CWM was never affected by the percentage of missing data for H, SLA, and LDMC. For SM, however, this ranking was affected as soon as 15% of the data had to be imputed with the relationships method (Fig. 4A2). When the missing data were replaced using the MICE method, only the ranking for SM was affected by the imputation (from 14% of missing data upwards; Table 3).

**Table 3:** Percentage of missing data at which the *P* value of the correlation between the ranking of the communities calculated without missing data and with imputed data became not significant for five of the 100 sets of communities, using the MICE, the dissimilarity or the Relationships imputation methods, or the deletion of species with one missing trait value

	Methods of imputation, resp. deletion			
	MICE	Dissimilarity	Relationships	Deletion
Functional identity				
H	\	45	\	11
LDMC	\	43	\	6
SM	14	31	15	10
SLA	\	42	\	7
Functional range					
H	39	40	45	14
LDMC	\	\	\	33
SM	17	32	12	7
SLA	\	\	\	23
Functional divergence					
H	\	25	33	10
LDMC	\	31	37	8
SM	\	40	5	7
SLA	\	32	37	10

H, vegetative height; LDMC, leaf dry matter content; MICE, Multivariate Imputation by Chained Equations; SM, seed mass; SLA, specific leaf area.

#### Functional range

With the imputation of missing data using the dissimilarity method, the ranking between grasslands on the functional ranges of SLA and LDMC was never affected by the percentage of missing data. For SM and H, this ranking was affected by a percentage of 32%, respectively, 40%, or more of missing data (Fig. 4B1). With the imputation of missing data using the relationships method, the ranking between grasslands for the functional range of SLA and LDMC was never affected by the percentage of missing data. For H, the ranking was only significantly affected by missing data when 45% of data were missing, while for SM, it was affected as soon as 12% of the data were missing (Fig. 4B2). Imputation with the MICE method led to different ranking between the grasslands compared to the ranking obtained with the original database starting 39% of missing data for H and 17% for SM, while the ranking was not influenced by the percentage of missing data for SLA and LDMC (Table 3).

#### Functional divergence

The R Pearson's coefficient between functional divergence indices calculated without missing data and with data imputed with single imputation decreased faster with increasing percentage of missing data as for the functional identity of functional range indices (Fig. 4). With imputation using the dissimilarity method, the ranking between grasslands for the functional divergence of SLA, LDMC, and SM was affected by the percentage of missing data from 31% to 40% of missing data upwards. The functional divergence of H was affected by the percentage of missing data starting 25% of data missing (Fig. 4C1). With imputation using the relationships method, the ranking between grasslands for the functional divergence of H, SLA, and LDMC was affected by the percentage of missing data when 33–37% or more of the data were missing. The functional divergence of SM was affected by the imputation already starting 5% of missing data (Fig. 4C2). With the MICE method, the ranking of the grasslands based on the divergence indices was not affected by the percentage of missing data (Table 3).

#### Deletion of species with missing trait values

The ranking between communities was quickly affected by the deletion of species with missing trait values for the four functional traits studied: 8% of missing data for CWM, 19% for functional range, and 9% for the functional divergence in average over the four traits (Table 3).

### Discussion of the effects of the imputation methods on functional diversity indices

The results clearly show the superiority of the tested imputation methods over the deletion of species with missing trait values for the estimation of functional diversity indices of grassland communities. They also show that single imputation methods that can be interpreted in ecological terms or Multivariate Imputation by Chained Equations can be used to replace missing data in a functional trait database to calculate functional diversity indices, with only few effects on the ranking between communities. None of these methods was able to perform best for all the traits and indices tested in this study. With the Multivariate Imputation by Chained Equations, the ranking of the grasslands was robust for all indices for the Height, the SLA, and the LDMC. But the accuracy of the MICE method was not better than the one of the single imputation methods based on ecological hypothesis for the functional identity and functional richness indices. For the Height, LDMC, and SLA, the relationship method performed as well that the MICE. For the SM, the dissimilarity method was the most accurate for the functional identity and range.

Consistently with the results at the species level, the distribution of the trait values seems to be a key parameter in explaining the robustness of the indices to imputation. Indeed, the indices calculated with the SM were more robust when imputation was conducted with the dissimilarity method. The SM exhibited an unbalanced distribution in the database with 606 species in contrast to the other traits. The results for the SM indicate that the MICE method also has to be used with caution for traits with an unbalanced distribution, although this was not obvious at the species level.

Using the dissimilarity method for the SM (unbalanced distribution) and the relationships method for the other traits (balanced distribution), the ranking between grasslands remained robust with up to 30% of the data missing for the functional identity (community-weighted mean), the functional range, as well as the functional divergence. We propose this percentage of missing data as a limit for the utilization of these single imputation methods. In our simulations, we randomly inserted the missing data by deletion. Each species had thus the same probability to have a missing value. The situation usually encountered in ecological studies is that the most common and dominant species have less missing data than the rare and subordinate species. Indices that are more influenced by dominant species than by minor ones (community mean value and function divergence) might therefore be, for the same percentage of missing data, less affected than in our study. For this type of indices, the 30% threshold is therefore conservative. In grassland plant communities, extreme trait values could be carried by dominant as well as by minor species, so that the effect of the repartition of the missing data is probably unsteady for the functional range index. The errors induced by the imputation of missing values has yet to be compared with other errors, such as those induced by the intraspecific variability of functional traits (Albert et al. 2010a,b2010b).

The 8–19% of missing data threshold for the deletion method cannot be compared with the 20% of abundance threshold propose by Garnier et al. (2004). Indeed, they proposed to measure the functional traits of dominant species only (no traits measured for the minor species). In our study, missing data occurred for both dominant and minor species and could affect one or several traits per species.

As discussed in the first part of this study, using the dissimilarity method might underestimate the functional distance between the species. We could therefore suppose that this method could be problematic previous to calculation of the functional range of the communities. However, the imputation was computed on the functional trait database with the 606 species. Species with extreme trait values in a community might not be functionally isolated in the database, so that the imputed values are not necessarily forced toward the median of the community. The ranking of the communities for their functional range was similarly affected by the percentage of replaced data with the dissimilarity as with the relationships or the MICE methods.

Multivariate functional diversity indices like those propose by Villeger et al. (2008) were not tested. Thus, the replacement method proposed here cannot be compared with the method of the Gower dissimilarity follow by a PCoA. However, Gower dissimilarity can only be computed between two species with at least one common trait documented and the PCoA can only be implemented if all the pairwise distances between species are known. This method will therefore only be useful for a low percentage of missing data or/and a large number of traits.

## Conclusions

At the species level, single imputation methods based on ecological hypothesis and multiple imputation by chained equations induced a lower error on the estimation of missing trait values than imputation by simple average or median computation. At the community level, the error induced by the replacement of missing values with single imputation methods based on ecological hypothesis or with multiple imputation by chained equations when calculating the functional identity, functional range, and functional divergence of plant communities is lower than that induced by omitting species with a missing value for a trait. The deletion of species with missing trait values or the utilization of simple imputation methods that do not take the functional differences between species into account (imputation by average or median values) should therefore be avoided prior to the computation of functional diversity indices using trait databases. Single imputation methods based on ecological hypothesis and adapted to the distribution of the trait values can be used instead of multiple imputations by chained equation when studying the ranking of communities for their functional diversity indices. The ranking of plant communities for these functional diversity indices was not significantly altered by imputing missing values with this method until 30% of the data were missing, as compared with calculation of the indices based on a database without missing data. For future research, improvement in the imputation of missing data in functional trait databases might be achieved by using ecological knowledge in multiple imputation methods.
